# Neurochemical Effects of 4-(2Chloro-4-Fluorobenzyl)-3-(2-Thienyl)-1,2,4-Oxadiazol-5(4H)-One in the Pentylenetetrazole (PTZ)-Induced Epileptic Seizure Zebrafish Model

**DOI:** 10.3390/ijms22031285

**Published:** 2021-01-28

**Authors:** Seong Soon Kim, Hyemin Kan, Kyu-Seok Hwang, Jung Yoon Yang, Yuji Son, Dae-Seop Shin, Byung Hoi Lee, Se Hwan Ahn, Jin Hee Ahn, Sung-Hee Cho, Myung Ae Bae

**Affiliations:** 1 Bio & Drug Discovery Division, Korea Research Institute of Chemical Technology, Daejeon 34114, Korea; firstsay@krict.re.kr (S.S.K.); khm4029@krict.re.kr (H.K.); kshwang@krict.re.kr (K.-S.H.); yjy1608@krict.re.kr (J.Y.Y.); yjson@krict.re.kr (Y.S.); dsshin@krict.re.kr (D.-S.S.); bnhlee@krict.re.kr (B.H.L.); 2 Department of Chemistry, Gwangju Institute of Science and Technology, Gwangju 61005, Korea; ansehwan@gist.ac.kr (S.H.A.); jhahn@gist.ac.kr (J.H.A.); 3 Chemical Analysis Center, Korea Research Institute of Chemical Technology, Daejeon 34114, Korea; 4 Department of Medicinal Chemistry and Pharmacology, University of Science & Technology, Daejeon 34113, Korea

**Keywords:** epilepsy, neurotransmitter, neurosteroid, metabolic alteration, zebrafish

## Abstract

Epilepsy is one of the most common neurological disorders, and it is characterized by spontaneous seizures. In a previous study, we identified 4-(2-chloro-4-fluorobenzyl)-3-(2-thienyl)-1,2,4-oxadiazol-5(4H)-one (GM-90432) as a novel anti-epileptic agent in chemically- or genetically-induced epileptic zebrafish and mouse models. In this study, we investigated the anti-epileptic effects of GM-90432 through neurochemical profiling-based approach to understand the neuroprotective mechanism in a pentylenetetrazole (PTZ)-induced epileptic seizure zebrafish model. GM-90432 effectively improved PTZ-induced epileptic behaviors via upregulation of 5-hydroxytryptamine, 17-β-estradiol, dihydrotestosterone, progesterone, 5α -dihydroprogesterone, and allopregnanolone levels, and downregulation of normetanephrine, gamma-aminobutyric acid, and cortisol levels in brain tissue. GM-90432 also had a protective effect against PTZ-induced oxidative stress and zebrafish death, suggesting that it exhibits biphasic neuroprotective effects via scavenging of reactive oxygen species and anti-epileptic activities in a zebrafish model. In conclusion, our results suggest that neurochemical profiling study could be used to better understand of anti-epileptic mechanism of GM-90432, potentially leading to new drug discovery and development of anti-seizure agents.

## 1. Introduction

Epilepsy is one of the most common neurological disorders, affecting approximately 65 million people. Epileptic seizures are characterized by complex behavioral phenotypes and electrical signals, culminating in neuronal cell death [1,2]. Previous studies have determined that the molecular mechanisms behind epileptic seizures are associated with imbalanced neurotransmission and abnormal neural activity [3,4].

Neurotransmitters function as chemical messengers that modulate emotion and behavior in animals [5,6]. Various neurotransmitter molecules, including dopaminergic, serotonergic, cholinergic, and GABAergic neurotransmitters, have been identified in brain tissue. GABAergic transmission is known as a key modulator of excitatory and inhibitory synaptic potentials in the nervous system. As the primary molecular mechanism underlying epileptic seizure is the inhibition of GABAergic signaling via gamma-aminobutyric acid type A (GABA_A_) receptors in vivo [7,8], the balance between excitatory and inhibitory signaling (E/I ratio) is especially important. A variety of anti-seizure drugs (ASDs) have focused on targeting GABA_A_ receptor function to regulate seizure activity [9,10]. In addition, numerous studies reported that pentylenetetrazole (PTZ) acts as an antagonist for GABA_A_ receptor with epileptogenic potential, the toxicological mechanism underlying epileptic seizures has been known to block the binding of GABA on the GABA_A_ receptors through in vivo practices [7,8].

Neurosteroids are endogenous steroids that act on the central nervous system (CNS). Neurosteroids affect neuronal function by binding to their respective intracellular receptors, which can then act as transcription factors to regulate gene expression [11,12]. In addition, neurosteroids modulate ligand-gated ion channels through non-genomic mechanisms. The 3α-reduced metabolites of progesterone (Prog) modulate GABA_A_ receptor ion channels [13,14,15], while other classical steroids, such as 17β-estradiol (E2), testosterone (T), and Prog, function as antagonists of the 5-hydroxytryptamine type 3 (5-HT_3_) receptor [16]. These ligand-gated channels comprise the majority of inhibitory neurotransmission in vertebrates. E2 has neuroprotective potential in neurodegenerative disease, and has been shown to contribute to the neuroprotective activity of anti-seizure drugs [17,18].

A number of animal models have been used to investigate the etiology of epilepsy and to identify anti-seizure drugs (ASDs) [19,20]. Among these models, the zebrafish (*Danio rerio*) is a promising vertebrate model for ASD screening, and has numerous advantages such as small cost, high fecundity, easy handling, light transparency, and high genetic homology to mammals [21,22,23].

Previously, we performed a phenotype-based screening of 6566 small molecules, using the zebrafish model. Among these small molecules, GM-90432 had drug potential as a novel anti-seizure drug (ASD) candidate, as it significantly alleviated seizure-like behavior in genetically- (*zc4h2* knockout in zebrafish larvae) or chemically- (PTZ treatment of mouse and zebrafish) induced epileptic seizure models. Furthermore, GM-90432 was blood–brain barrier (BBB)-permeable. The anti-epileptic effect was evaluated using p-ERK levels as a marker of neural activity, electroencephalograms (EEGs) as indicators of seizure-like events, and voltage-gated NA^+^ channel currents as measurements of neural excitability in zebrafish and hippocampal CA3 pyramidal neurons. However, the underlying anti-epileptic mechanism of GM-90432 remains unclear [24].

In the present study, to confirm the protective effect of GM-90432 against neurotoxicity induced by PTZ and the possibility of GM-90432 as an anti-seizure agent, we investigated metabolic alteration of neurotransmitters and neurosteroids induced by GM-90432, PTZ, or PTZ after treatment with GM-90432 in the brains of zebrafish. The levels of the neurotransmitters 5-hydroxytryptamine (5-HT; serotonin) and E/I ratios, and neurosteroids E2, Prog, 5α-dihydroprogesterone (5α-dihydroProg), and allopregnanolone (Allo-P) reduced upon exposure to PTZ were significantly restored in zebrafish treated with GM-90432 prior to PTZ. We also examined the protective effect of GM-90432 via reactive oxygen species (ROS) scavenging and overall survival in PTZ-induced oxidative stress in zebrafish larvae. While ROS were increased by PTZ, zebrafish larvae treated with GM-90432 prior to exposure to PTZ showed ROS levels similar to controls. These results indicate that GM-90432 exerts neuroprotective effects via improvement of neurotransmitters and neurosteroid imbalance, as well as ROS scavenging activity in a PTZ-induced epileptic seizure model in zebrafish.

## 2. Results

### 2.1. Metabolic Neurotransmitters Alterations

**Figure 1:**
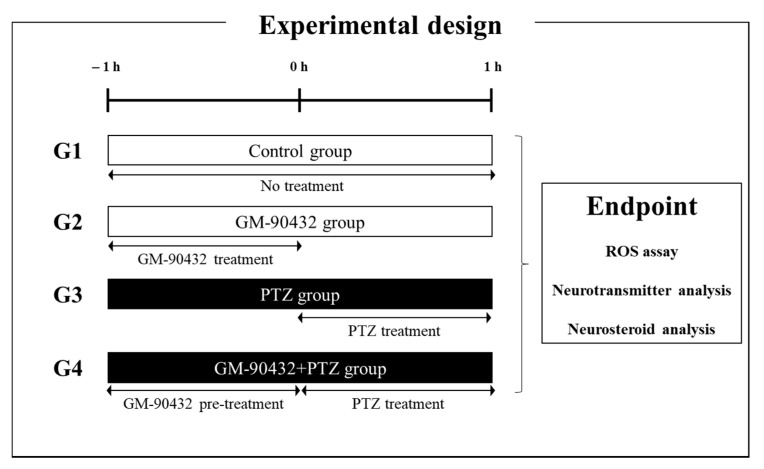
Experimental scheme used to determine the protective effect of GM-90432 against PTZ-induced epileptic seizures in zebrafish larvae and adults. Zebrafish were separated into 4 different treatment groups (G): G1: control group, G2: GM–90432 group, G3: PTZ-induced epilepsy model group, and G4: GM-90432 + PTZ group. At the experimental endpoint, zebrafish were sacrificed for analysis of ROS levels (larva only), and neurotransmitter and neurosteroid analyses.

**Figure 2:**
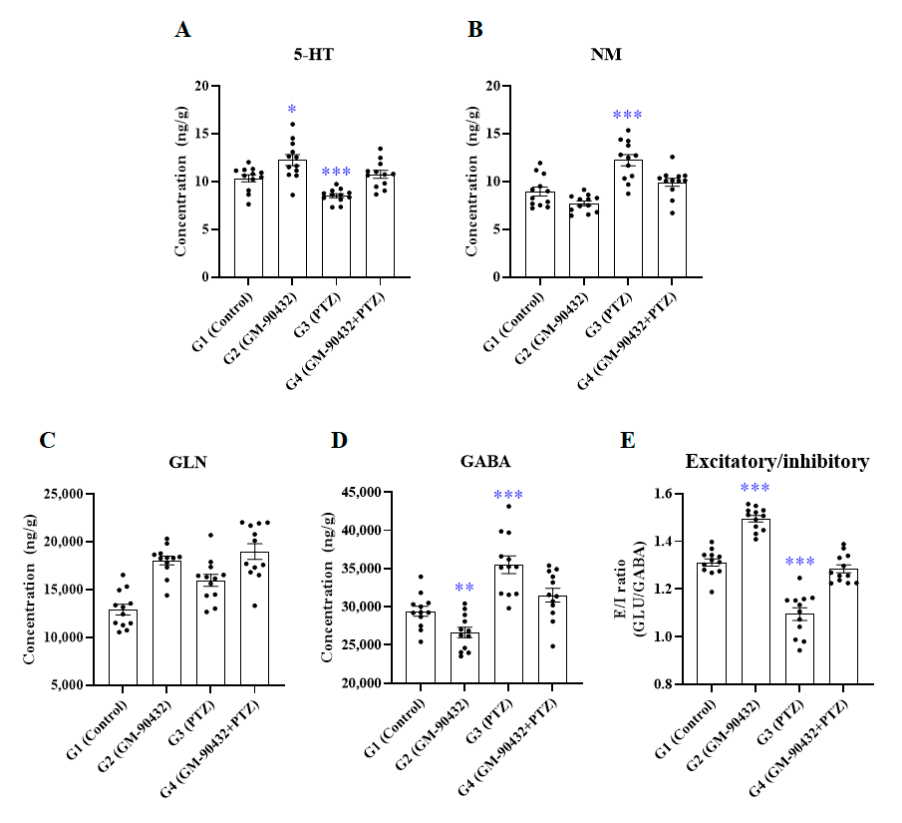
The levels of 5-HT (**A**), NM (**B**), GLN (**C**), and GABA (**D**), and excitatory/inhibitory ratio (**E**) in brain samples of zebrafish exposed to GM-90432, PTZ, or PTZ following pretreatment with GM-90432. Values represent means ± standard error of the mean (SEM) of 12 samples. Significance between control and exposure groups is indicated by * *p* < 0.05, ** *p* < 0.01, and *** *p* < 0.001.

To understand the mechanism of GM-90432-induced neuroprotection against PTZ-induced epileptic seizure, we focused on neurochemical metabolism in the brain tissue of zebrafish. First, adult zebrafish (*n* = 10) were acutely exposed to 1 or 5 mM PTZ for 1 h in a 1 L tank. One micromolar PTZ was selected as the optimal concentration of PTZ that had no observable effect (NOEC) on fish’s survival and morphological alteration for 24 h compared to controls (data not shown). Next, 2 µM GM-90432 was optimized as a non-toxic concentration that did not induce any morphological malformation or death in adult zebrafish. Thereafter, we investigated the neuroprotective effect of GM-90432 against PTZ-induced neurotransmitter imbalance in adult zebrafish (*n* = 12) according to experimental design (Figure 1 and Table S1). A variety of neurotransmitter alterations in amino acid, cholinergic, dopaminergic, serotonergic, and GABAergic systems were quantified using LC-MS/MS in brain tissue (Figure 2 and Table S1). The PTZ-treated group (G3) showed various neurotransmitters alterations compared to the control group; acute exposure to PTZ for 1 h significantly decreased 5-HT (t_(22)_ = 4.354; *p* < 0.0005) and increased NM (t_(22)_ = 4.323; *p* < 0.0005) and GABA (t_(22)_ = 4.625; *p* < 0.0001) levels in brain tissue, compared to the control group (G1). On the contrary, the GM-90432-treated group (G2) displayed increased 5-HT (t_(22)_ = 2.790; *p* < 0.02) and decreased NM (t_(22)_ = 2.350; *p* < 0.03) and GABA (t_(22)_ = 2.975; *p* < 0.01) levels compared to G1. GM-90432 pretreatment followed by exposure to PTZ for 1 h (G4) significantly alleviated PTZ-induced neurotransmitters alteration, as altered levels of 5-HT (t_(22)_ = 4.946; *p* < 0.0001), NM (t_(22)_ = 3.187; *p* < 0.005), and GABA (t_(22)_ = 2.726; *p* < 0.02) were significantly attenuated compared to G3 in the brain tissue of adult zebrafish. However, GM-90432-treated group and PTZ-treated group did not altered GLN level, compared to the control group (Figure 2C).

One-way ANOVA with Tukey’s post hoc test were conducted to examine the statistical significance among groups (Table S2). The levels of 5-HT (F_(3,44)_ = 14.1; *p* < 0.0001), NM (F_(3.44)_ = 18.0; *p* < 0.0001), and GABA (F_(3, 44)_ = 18.5; *p* < 0.0001) were found to be significantly different, but not altered GLN (F_(3,44)_ = 0.9946; *p* < 0.4042) (Figure 2A–D). Furthermore, we examined the excitatory and inhibitory (E/I) ratio, calculated from GLU and GABA levels in brain tissue. The data showed that G3 displayed a significantly decreased E/I ratio (t_(22)_ = 7.076; *p* < 0.0001), while G2 displayed a significantly increased E/I ratio, compared to G1 (t_(22)_ = 8.798; *p* < 0.0001). In addition, G4 displayed a dramatically attenuated E/I ratio compared to G2 (t_(22)_ = 6.081; *p* < 0.0001) (Figure 2D), suggesting that GM-90432 rebalanced 5-HT, NM, and GABA levels as wells as E/I ratios in PTZ-induced neurotransmitters imbalanced brain tissues in adult zebrafish.

### 2.2. Metabolic Neurosteroid Alterations

**Figure 3:**
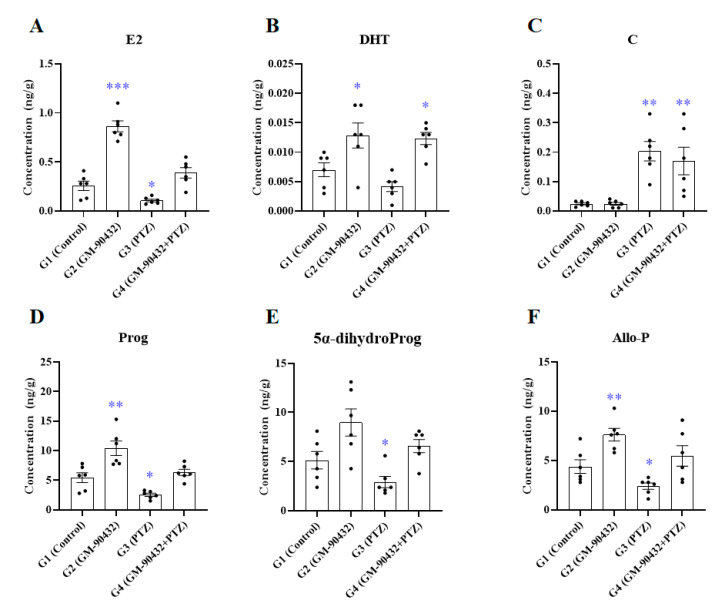
The levels of E2 (**A**), DHT (**B**), C (**C**), Prog (**D**), 5α-dihydroProg (**E**), and Allo-P (**F**) in brain samples of zebrafish exposed to GM-90432, PTZ, or PTZ following pretreatment with GM-90432. Values represent means ± SEM of 6 samples. The brain samples from 2 individual zebrafish were pooled to form composite samples. Significance between control and exposure groups is indicated by * *p* < 0.05, ** *p* < 0.01, and *** *p* < 0.001.

To investigate alterations in metabolic neurosteroids induced by GM-90432, PTZ, or PTZ after pretreatment with GM-90432 compared to controls, the levels of neurosteroids in the brains of zebrafish were analyzed by LC-MS/MS (Figure 3 and Table S3). In zebrafish exposed to 1 mM PTZ for 1 h (G3), the levels of E2 (t_(10)_ = 3.035; *p* < 0.04), Prog (t_(10)_ = 3.313; *p* < 0.03), 5α-dihydroProg (t_(10)_ = 2.098; *p* < 0.03), and Allo-P (t_(10)_ = 2.595; *p* < 0.02) were all decreased compared with the control group G1. On the other hand, the levels of C (t_(10)_ = 5.375; *p* < 0.004) were significantly increased. The levels of E2 (t_(10)_ = 8.295; *p* < 0.001), DHT (t_(10)_ = 2.403; *p* < 0.03), Prog (t_(10)_ = 3.309; *p* < 0.01), and Allo-P (t_(10)_ = 3.405; *p* < 0.004) in zebrafish treated with GM-90432 for 1 h (G2) were significantly increased compared to G1. The levels of E2, Prog, 5α-dihydroProg, and Allo-P that were reduced when exposed to PTZ were rescued by GM-90432 pretreatment (G4), and were not significantly different compared to G1. The levels of DHT increased by treatment with GM-90432 (G2) were still increased compared to the control group even after exposure to PTZ after treatment with GM-90432 (t_(10)_ = 3.411; *p* < 0.03). The levels of C in zebrafish exposed to PTZ after pretreatment with GM-90432 were increased compared to the control group (t_(10)_ = 3.102; *p* < 0.03), similar to the group exposed to PTZ. One-way ANOVA with Tukey’s post hoc test was performed to confirm significant differences in metabolic neurosteroid levels between groups (G1 to G4, Table S4). The levels of E2 (F_(3, 20)_ = 51.4; *p* < 0.0001), DHT (F_(3, 20)_ = 9.282; *p* < 0.0005), C (F_(3, 20)_ = 10.76; *p* < 0.0002), Prog (F_(3, 20)_ = 16.28; *p* < 0.0001), 5α-dihydroProg (F_(3, 20)_ = 7.521; *p* < 0.002), and Allo-P (F_(3, 20)_ = 9.067; *p* < 0.0005) were significantly altered between groups. 

**Figure 4:**
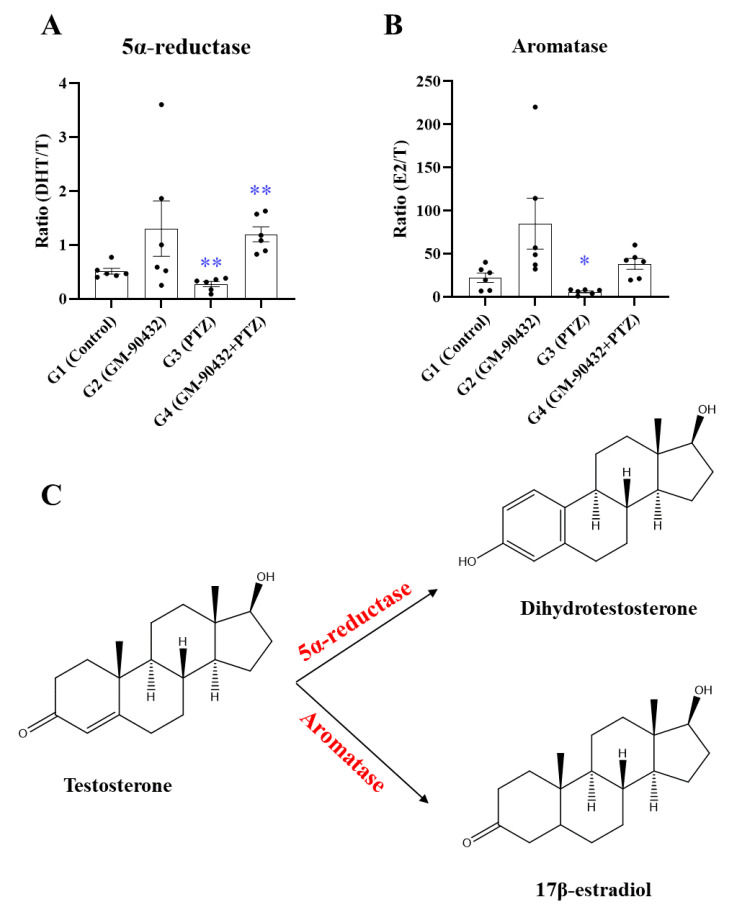
The enzymatic activity by altered metabolic ratio in brain samples of zebrafish. (**A**) Altered metabolic ratio (5α-reductase) of T to DHT in brain samples of zebrafish exposed to GM-90432, PTZ, or PTZ following pretreatment with GM-90432. (**B**) Altered metabolic ratio (aromatase) of T to E2 in brain samples of zebrafish exposed to GM-90432, PTZ, or PTZ following pretreatment with GM-90432. (**C**) The metabolic pathway of T conversion to DHT or E2. Values represent means ± SEM of 6 samples. Brain samples from 2 individual zebrafish were pooled to form composite samples. Significance between control and exposure groups is indicated by * *p* < 0.05 or ** *p* < 0.01.

To examine enzymatic activities involved in steroid metabolism, the ratios of steroid metabolites to precursors were investigated (Figure 4C). The metabolic ratio of T to DHT, which reflects 5α-reductase enzymatic activity, was significantly decreased in the brains of zebrafish after exposure to PTZ compared to controls (t_(10)_ = 3.291; *p* < 0.01, Figure 4A). On the other hand, this activity was increased in the brains of zebrafish exposed to PTZ following pre-treatment with GM-90432 (t_(10)_ = 4.621; *p* < 0.001, Figure 4A). The metabolic ratio of T to E2, which reflects aromatase enzymatic activity, was also significantly decreased after exposure to PTZ compared to controls (t_(10)_ = 2.917; *p* < 0.05, Figure 4B). However, this activity was not rescued by pretreatment with GM-90432.

### 2.3. Protective Effect of GM-90432 on PTZ-Induced ROS Generation and Zebrafish Death

We investigated the protective effect of GM-90432 against PTZ-induced ROS generation and death in zebrafish larvae. In a preliminary study, we determined the optimal concentration of PTZ in zebrafish larvae; among tested concentration, 5 mM PTZ demonstrated the highest ROS generation after 1 h exposure, and highest amount of larval death after 24 h exposure. Therefore, we performed two experiments using different exposure times, 1 h for ROS detection or 24 h for survival analysis. 

**Figure 5:**
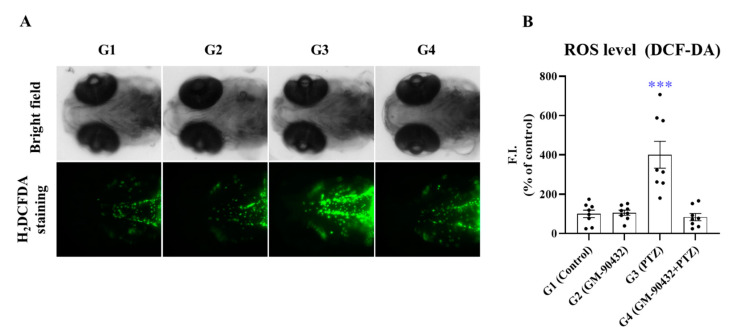
Protective effect of GM-90432 in PTZ-induced ROS generation. (**A**) The intracellular ROS were stained with DCF-DA dye for 20 min. Fluorescence images of ROS generation in zebrafish larvae in the 4 experimental groups. (**B**) The relative fluorescence intensities of ROS levels in individual larvae were quantified. Values represent means ± SEM of 10 samples. Significance between control and exposure groups is indicated by *** *p* < 0.001.

Our data showed that ROS was generated in most brain regions, such as fore brain and tectum, in G3 zebrafish. ROS levels increased by 400% (t_(14)_ = 4.236; *p* < 0.001, Figure 5A), and survival rate decreased by 10% (t_(4)_ = 9.000; *p* < 0.001, Figure 5B) compared to G1, likely due to enhanced oxidative stress in zebrafish larvae. However, GM-90432 pretreatment prior to PTZ treatment suppressed ROS generation (t_(14)_ = 4.483; *p* < 0.0005, Figure 5B), and different concentrations of GM-90432 (2.5 to 10 μM) increased survival rate in a dose-dependent manner (F_(4, 10)_ = 42.13; *p* = 0.0001, Figure S1) in zebrafish larvae. Therefore, our results suggest that GM-90432 has a neuroprotective effect against PTZ-induced oxidative stress and death via inhibition of ROS generation in zebrafish larvae.

## 3. Discussion

We have previously published the results of a phenotype-based ASD screen of 6566 small molecules, including a variety of pharmacophores, in chemically- or genetically-induced models of epilepsy. Among these small molecules, we identified GM-90432 as a novel pharmacophore for treatment of epileptic seizure, and its pharmacological properties were evaluated in vitro and in vivo. GM-90432 effectively reduced seizure-like behaviors, and regulated EEG and neural activity in PTZ-induced epileptic seizure mouse and zebrafish models. Furthermore, the pharmacokinetic assessments suggested that GM-90432 could be used in animal models [24]. However, the underlying anti-epileptic mechanism of GM-90432 remained unclear.

Numerous studies have suggested that zebrafish are a promising model for epileptic seizures and ASD screening [25,26,27]. The PTZ-induced zebrafish seizure model shares similar characteristics with mammals, including abnormal locomotor and electroencephalographic (EEG) activity and upregulation of c-fos expression, a biomarker of epileptic seizure [28,29,30].

In the present study, we focused on a neurochemical profiling-based approach to understand the anti-epileptic effects of GM-90432 in a PTZ-induced epileptic seizure zebrafish model. We examined the modulating effects of GM-90432 on a variety of neurotransmitters and neurosteroids in zebrafish brain tissue. Neurotransmitters and neuroactive amino acids are closely related to neural network and behavioral profiles, and imbalances in these neurotransmitters at the presynapse or postsynapse may contribute to neurological disorders such Alzheimer’s disease, Parkinson’s disease, or seizure [31,32,33]. Therefore, five categories of neurotransmitters (amino acids, and cholinergic, dopaminergic, serotonergic, and GABAergic ligands) were quantitatively analyzed by LC-MS/MS. Among these molecules, we found that 5-HT, NM, and GABA levels were significantly altered, but GLN level was not significantly modulated in brain tissue of adult zebrafish by PTZ exposure, and these changes were markedly rescued by GM-90432 pretreatment (Figure 2A–D).

In amino acid ligand, previous studies reported that amino acid profiles are altered during epileptic activity in both plasma and cerebrospinal fluid (CSF) of epileptic patients [34,35,36]. However, GM-90432 and PTZ co-treatment (G4) did not show significant changes compared to the PTZ-treated group (G3) in zebrafish. Therefore, we could not determine a pharmacological relationship between neuroactive amino acids and GM-90432 treatment in a zebrafish model of epilepsy.

In cholinergic ligand, several studies have demonstrated that antagonists of muscarinic and nicotinic acetylcholine receptors (nAChRs) reduced seizures in rodent models [37,38]. Furthermore, cholinergic dysfunction affects neuronal excitability and temporal lobe epilepsy in epileptic patients [39]. Previous animal studies have reported that ACHO and CHO were increased in the cortex and hippocampus, but not in the striatum, due to cholinergic receptor activation in epileptic seizure models [40]. On the contrary, our data showed that ACHO levels were increased in PTZ, GM-90432, or co-treatment with PTZ and GM-90432 groups (G2, G3, and G4) compared to controls. Additionally, CHO levels were increased in PTZ alone or PTZ and GM-90432 co-treated zebrafish, compared to controls. Therefore, our results indicated that GM-90432 could not improve cholinergic imbalance as a result of PTZ-induced neurotransmitters alterations in the zebrafish brains.

In dopaminergic ligand, several reports have described the role of DA signaling in modulating seizures, suggesting an inhibitory activity on hippocampal excitability via activation of DA receptors [41,42]. Furthermore, dopaminergic dysfunction has been detected in both epileptic human brains and animal seizure models. Increased DA levels and firing of DA neurons were observed in temporal lobe epilepsy in rodent models [43,44]. In this study, our data showed that PTZ exposure significantly increased the DA synthesis pathway in zebrafish, as indicated by PHE to TYR conversion, but this did not lead to increased DA levels compared to the control group (Table S1); additionally, GM-90432 co-exposure with PTZ did not attenuate these increased levels compared to PTZ-exposed zebrafish. However, PTZ exposure markedly increased levels of NM, which is a DA metabolite, and GM-90432 co-treatment with PTZ decreased NM to levels similar to controls (Figure 2C). There are few studies on the role of NM as a chemical biomarker, but NM was identified as upregulated in seizure or stroke patients [45,46]. Therefore, our results, which increased NM levels were found in the brain tissue of PTZ-exposed adult zebrafish, were supported by previous findings. We suggest that the neuroprotective effect of GM-90432 against PTZ-induced neurotransmitter alteration may be associated with NM metabolism, but not with the DA synthesis pathway in zebrafish.

In serotonergic ligand, seizure modulating activity of TRYP, 5-HT, and 5-HTP on the anti-epileptic effects of anti-depressant drugs has been reported in both human and animal studies [47,48,49]. These findings demonstrated that 5-HT depletion increased seizure susceptibility and neuropathological disorders, and the anti-epileptic effects of drugs were abolished by deficiency of a postsynaptic 5HT receptor subtype. These works suggested that low concentration of 5-HT could be a potential risk factor for epilepsy. In this study, PTZ-exposed zebrafish displayed significantly decreased 5-HT levels compared to controls, and these decreased 5-HT levels were rescued by GM-90432 co-treatment. Therefore, our results demonstrate GM-90432 may have biphasic neuroprotective potential, through serotonergic-mediated anti-epileptic effects and anti-depressant activity.

In the GABAergic ligand, the GABA_A_ receptor has historically been the primary target of most anti-epileptic agents. In general, the GABA_A_ receptor is known as the predominant inhibitory neurotransmitter receptor in the brain. The activation of GABA_A_ receptor allows the influx of Cl^−^, which leads to inhibition of neuronal activity. In this study, PTZ, an antagonist of the GABA_A_ receptor, was used to induce neurotransmitters imbalances in a zebrafish model. PTZ exposure led to increased levels of GABA and GLN, but not of GLU, compared to the control group. PTZ exposure also induced disruption of E/I balance, calculated by GLU/GABA levels, resulting in neural activation in the brain tissue of adult zebrafish compared to the control group. In general, the balance between excitatory GLU and inhibitory GABA neurotransmitters modulates excitatory and inhibitory conductance, affecting seizure activity in brain tissue [50]. We found here that GM-90432 co-treatment with PTZ clearly attenuated the PTZ-induced accumulation of GABA, leading to restoration of the E/I ratio to a similar level as the control group. This result suggested that the GABAergic modulating effect of GM-90432 may contribute to anti-epileptic mechanisms in zebrafish.

Steroid hormones, which are produced in various endocrine organs, easily cross the BBB because of their lipophilic nature [51,52]. Circulating steroid hormones serve as precursors for the synthesis of neurosteroids, which are steroid hormones formed within the brain, as enzymes typically produced in steroidogenic tissues are also found in the nervous system [53,54]. Neurosteroids can also be synthesized in the brain itself without the aid of peripheral resources. Neuroactive steroids modulate neuronal function by both genomic (as in classical intracellular steroid receptors) [11,12] and non-genomic rapid actions (as in ion channels and membrane receptors) [13,15] in the brain. When zebrafish were treated with PTZ, an antagonist of the GABA_A_ receptor, the levels of Prog and its metabolites 5α-dihydroProg and Allo-P were decreased (Figure 3D–F). Prog and its metabolites positively modulate GABA_A_ receptors by putatively binding at distinct sites of the α subunit and at the α/β subunit interface [55]. Allo-P has shown anticonvulsant activity in diverse preclinical models [56,57,58,59], and protected rats from chemoconvulsant-induced seizures in a model of refractory status epilepticus, where seizures are unremitting, in a dose-dependent manner [60]. The levels of Prog and its metabolites were increased by GM-90432 treatment, and co-treatment of GM-90432 and PTZ effectively restored these steroids to the levels of the control group (Figure 3D–F). The steroid hormone E2 inhibits N-methyl-D-aspartate (NMDA)-induced whole cell currents and NMDA-stimulated increase in intracellular free calcium, and protects against NMDA-induced neuronal death [61]. Oxidative stress, and lipid peroxidation in particular, plays a significant role in neuronal death initiated by NMDA receptor activation [62,63]. Neuroprotection via E2 is mediated by direct inhibition of NMDA-stimulated calcium entry. Furthermore, E2 is a potent antioxidant, and reduces the levels of free radicals [64] and apoptosis via increased production of antiapoptotic proteins Bcl-2 and Bcl-xL [65,66]. The levels of E2 that were reduced upon exposure to PTZ were increased by GM-90432 co-treatment to the level of the control group (Figure 3A). These results indicate that GM-90432 reduces PTZ-induced neurotoxicity through increased levels of E2, Prog, 5α-dihydroProg, and Allo-P.

Enzyme activities, which can be monitored by measuring the reactions between target enzymes and substrate molecules using radioimmunoassays and/or enzyme immunoassays, describe the functional diversity of biological systems, which is driven by genetic diversity. However, these conventional methods have several disadvantages, such as overestimating cross-reacting antibodies, which limits the applicability of these assays; in addition, the activity of only one enzyme at a time can be examined. In contrast, estimating enzyme activities by examining the ratios of metabolites to precursors based on chromatography-mass spectrometry has better quantitative reproducibility and can be used to investigate broader enzyme activity profiles [67]. In current study, two enzymatic activities, 5α-reductase (the metabolic ratio of T to DHT) and aromatase (the metabolic ratio of T to E2) were calculated to be reduced upon exposure to PTZ, and then increased by GM-90432 co-treatment (Figure 4). The activities of 5α-reductase and aromatase are localized in the temporal and in the frontal brain areas, including the cerebral neocortex, subcortical white matter, and hippocampus [68]. A previous study reported that the reduction of T by 5α-reductase decreased brain excitability via activation of the GABA_A_ receptor [69]. Therefore, enzymes regulating the metabolism of T may be targets of novel strategies for the development of new ASDs.

Increasingly, studies have emphasized that oxidative stress is associated with the initiation and progression of epilepsy, and that excessive ROS could be a key factor in seizure-induced neuronal damage [70,71,72,73]. In addition, several studies have demonstrated that PTZ is an inducer or ROS, and triggers excessive free radical generation and suppression of the cellular antioxidant defense system, leading to oxidative stress in animal seizure models [74,75]. Several other studies describe the toxicological relationship between oxidative stress and epilepsy in animals. Brain tissue is very sensitive to excessive free radicals, which can induce neurological disorders including seizures, subsequently leading to neuronal cell death in vitro and in vivo. Our data showed that GM-90432 markedly suppressed ROS generation and increased survival rate in PTZ-induced oxidative stress in zebrafish larvae (Figure 5 and Figure S1). These data suggest that GM-90432 may exhibit neuroprotective potential against PTZ-induced ROS generation and epileptic seizure induction via ROS scavenging in zebrafish.

In this study, we performed neurochemical profiling analysis in brain tissue to better understand the neuroprotective mechanism of GM-90432 against PTZ-induced epileptic seizure in zebrafish. We showed that GM-90432 exerts multiple neuroprotective effects via correction of the expression of a variety of endogenous neurotransmitter and neurosteroid molecules disrupted by PTZ treatment. Furthermore, GM-90432 showed protective effects against PTZ-induced oxidative stress and zebrafish death, suggesting that GM-90432 may exhibit biphasic potential via antioxidant and anti-epileptic activities. Although our study did not elucidate the molecular mechanism of the anti-epileptic effect, our data suggest that GM-90432 exerts this anti-epileptic effect via upregulation of 5-HT, E2, DHT, Prog, 5a-dihydroProg, and Allo-P levels, and downregulation of NM, GABA, and C levels, in addition to the E/I ratio in PTZ-induced epileptic seizure zebrafish model. In addition, our study provides toxicological information about the PTZ-induced disruptions of neurotransmitters and neurosteroid levels in brain tissue, by analyzing the endogenous profiles to establish the epileptic patterns in brain tissue of this animal model. Finally, these neurochemical profiling approaches could be used for new drug development and discovery of anti-seizure agents.

## 4. Materials and Methods

### 4.1. Materials

GM-90432 (≥99% purity) was synthesized by the Center for Medicinal Chemistry at the Gwangju Institute of Science and Technology, Gwangju, Korea. Neurotransmitters standards including serotonergic ligands tryptophan (TRYP), 5-hydroxy tryptophan (5-HTP), 5-Hydroxytryptamine (5-HT), 5-hydroxyindoleacetic acid (5-HIAA), melatonin (ME), kynurenergic ligands kynurenine (KYN), kynurenic acid (KYNA), anthranilic acid (AA), 3-hydroxykynurenine (3-HK), 3-hydroxyanthranilic acid (3-HAA), dopaminergic ligands phenylalanine (PHE), tyrosine (TYR), 3,4-dihydroxy-L-phenylalanine (L-DOPA), dopamine (DA), norepinephrine (NE), epinephrine (E), 3-methoxytyramine (3-MT), normetanephrine (NM), tyramine (TA), cholinergic ligands acetylcholine (ACHO), choline (CHO), betaine (BET), serine (SE), and GABAergic ligands gamma-aminobutyric acid (GABA), glutamic acid (GLU), glutamine (GLN), histamine (HA), histidine (HIS), leucine (LEU), and lysine (LYS) were purchased from Sigma-Aldrich (St. Louis, MO, USA). Isotope-labeled TRYP-*d*_3_, GLU-*d*_5_, and L-DOPA-*d*_6_ were purchased from CDN ISOTOPE (Pointe-Claire, Quebec, Canada). Neurosteroids including E2, T, dihydrotestosterone (DHT), cortisol (C), Prog, 5α-dihydroProg, and Allo-P were obtained from Sigma-Aldrich Corporation or Steraloids, Inc. (Newport, RI, USA). Internal standards 2,4,16,16,17-d_5_-estradiol for E2, dehydroepiandrosterone-2,2,3,4,4,5-d_6_ for T and DHT, 9,11,12,12-d_4_-cortisol for C, and pregnenolone-17β,21,21,21-d_4_ for Prog, 5α-dihydroProg, and Allo-P were purchased from Sigma-Aldrich Corporation and CDN Isotopes, Inc. (Pointe-Claire, Quebec, Canada). For the solid-phase extraction [7], an Oasis PRiME Hydrophilic-Lipophilic Balanced (HLB) cartridge (1 mL, 30 mg; Waters Corporation, Milford, MA, USA) was preconditioned with 1 mL of methanol (MeOH) followed by 1 mL of deionized water. All organic solvents were analytical or HPLC grade, and were purchased from J.T. Baker (Phillipsburg, NJ, USA). The deionized water was prepared using the Milli-Q purification system (MilliporeSigma, Burlington, MA, USA).

### 4.2. Animals Care and Chemical Treatment

Wild type adult male zebrafish (12 months old) were kept under a 14/10 h light/dark cycle according to standard zebrafish breeding and maintenance protocol [76]. The fish were and fed three times a day with brine shrimp. All animal experiments were performed in accordance with the NIH guide for the care and use of Laboratory Animals (No.8023, revised in 1996), and this work was approved by the Animal Care and Use Committee of the Korea Research Institute of Chemical Technology.

Adult male fish were used in both neurotransmitter and neurosteroid analyses. Zebrafish embryos were obtained from natural mating, and healthy embryos were incubated in embryo medium refreshed daily until 5 days post-fertilization (dpf) at 28 °C. Only 5 dpf zebrafish larvae were used for survival and ROS assays. As it was previously reported that GM-90432 reached steady-state after 1 h exposure [24], both larval and adult zebrafish were separated into 4 different treatment groups (Figure 1): Group 1 (G1, control group) fish were exposed to DMSO at a similar concentration as test compounds; Group 2 (G2, GM-90432 group) fish were exposed to GM-90432 (10 µM for larvae, or 2 µM for adult zebrafish); Group 3 (G3, PTZ-induced epilepsy model group) fish were exposed to PTZ (5 mM for larvae, or 1 mM for adult zebrafish); and Group 4 (G4, GM-90432+PTZ group) fish were exposed to PTZ following 1 h GM-90432 pretreatment. The experiments were conducted in 24 well plates (for larval fish) or 1 L tanks (for adult zebrafish), respectively.

### 4.3. Analysis of Neurotransmitters in the Brain of Zebrafish

Brain tissues (*n* = 12) were added to nine volumes (*w*/*v*) of pre-cooled distilled water and homogenized with a probe sonicator (Ultrasonic processor VCX-130, Sonics & Materials Inc, Newtown, CT, USA) on ice. Each homogenate was mixed with an equal volume of ice-cold MeOH containing ISs with 1% formic acid, and followed by vortexing and centrifugation for 10 min at 4 °C. Supernatants were filtered through 0.2 µm GH Polypro (GHP) filter plates (Acro PrepTM, Pall Corporation, Ann Arbor, MI, USA) and stored at −20 °C until analysis by LC-MS/MS. Thirty-one neurotransmitters representing five different categories were separated by linear gradient elution at 0.2 mL/min using 0.1% formic acid in water (eluent A) and 0.1% formic acid in MeOH (eluent B) according to the following gradient mode: beginning at 0% B from 0 to 0.5 min, increasing to 20% B at 2.5 min, increasing to 90% B at 13.0 min, remaining constant for 13.5 min, and then re-equilibrating to the initial condition over 17.0 min. Chromatographic separation was conducted using a pentafluorophenyl (PFP) column (100 mm × 2.1 mm i.d., 1.9 μm; YMC, JAPAN) with a PFP guard column (2.1 mm × 5 mm i.d., 1.9 µm; YMC) at 40 °C. Injection volume was 3 μL. Multiple reaction monitoring (MRM) mode by electrospray positive ionization (ESI+) was used for neurotransmitters quantification. The optimized mass parameters were set as follows: source temperature of 150 °C, desolvation temperature of 550 °C, cone gas flow 150 L/h, desolvation gas flow of 1000 L/h, cone gas flow of 150 L/h, and collision gas flow of 0.15 mL/min.

### 4.4. Analysis of Neurosteroids in the Brains of Zebrafish

The brain samples (*n* = 6) from 2 individual zebrafish were pooled to form a composite sample, and were prepared as previously described [77,78], with some modification. Briefly, 10 µL of the ISs mixture was added to the samples to a concentration of 50 ng/mL, and then samples were homogenized in 1 mL of MeOH/acetic acid (99:1 *v*/*v*). After an overnight extraction at 4 °C, the samples were centrifuged at 12,000 rpm for 5 min, and the pellet was extracted twice with 1 mL of MeOH/acetic acid (99:1 *v*/*v*). The organic phases were combined and dried under a stream of nitrogen. The samples were resuspended with 1 mL of MeOH/water (10:90 *v*/*v*). The mixture was extracted with Oasis PRiME HLB SPE cartridges coupled to a peristaltic pump. After loading a sample onto a cartridge, the cartridge was washed with 1 mL of water and eluted twice with 1 mL of MeOH. The combined MeOH eluates were evaporated under a stream of nitrogen, and then dissolved with 100 µL of MeOH. Finally, 5 µL of this solution was injected into the LC-MS/MS system.

LC-MS/MS analysis was performed using a Waters^®^ Acquity UPLC I-Class system (Waters Corporation) that interfaced with a Waters^®^ Xevo TQ-S micro tandem mass spectrometer (Waters Corporation) with electrospray ionization (ESI) source [77,79]. Chromatographic separation was achieved with a Waters^®^ Acquity UPLC BEH C_18_ octadecylsilane column (2.1 mm × 100 mm, 1.7 µm). The LC conditions for the separation were mobile phase A was 0.2 mM ammonium fluoride in H_2_O, and mobile phase B was 0.2 mM ammonium fluoride in MeOH. The gradient program (*v*/*v*) had a flow rate of 400 µL/min, and was as follows: the program began with 30% B (*v*/*v*), held at 30% B (*v*/*v*) for 5 min, then increased to 50% B (*v*/*v*) at the 15 min mark, and held at 50% B (*v*/*v*) for 5 min, then increased to 55% B (*v*/*v*) at the 23 min mark, then increased to 80% B (*v*/*v*) at the 27 min mark, and held at 80% B (*v*/*v*) for 3 min. The column was re-equilibrated over 3 min with 30% B (*v*/*v*). The temperature of the column was maintained at 40 °C. Separated steroid hormones were monitored by positive (for T, DHT, C, Prog, 5α-dihydroProg, and Allo-P) and negative (for E2) electrospray ionization (ESI) tandem mass spectrometry (MS/MS). The source and operating parameters were optimized as follows: capillary voltage of 3 kV (ESI^+^) and 4 kV (ESI^−^), cone gas flow of 30 L/h, desolvation temperature of 450 °C, and desolvation gas flow of 800 L/h. Quantitative analysis was performed in MRM mode, and peak identifications were achieved by comparing retention times and matching the MS/MS ions. Data acquisition was performed using MassLynx software (V4.1, Waters Corporation).

### 4.5. Reactive Oxygen Species and Survival Assays

To examine the protective effect of GM-90432 on PTZ-induced ROS generation and zebrafish death, 5 dpf zebrafish larvae were pretreated with 10 µM GM-90432 for 1 h, and then fish were exposed to 5 mM of PTZ for either 1 h (to examine the ROS scavenging effect, *n* = 8) or 24 h (to measure the survival rate, *n* = 10, triplicates).

For live imaging and quantification of ROS, fish were stained with H2DCFDA (Invitrogen, Carlsbad, CA, USA) for 20 min, then washed twice with embryo medium. Zebrafish larvae were anesthetized in 0.016% Tricaine, and the brain regions were observed with a Lionheart FX automated microscope (BioTek, Winooski, VT, USA) running Gen5 Image Prime software (Ver. 3.08). The fluorescence intensities were quantified using Image J software. For measurement of survival rate, fish were visually monitored for morphology and heart beat (cardiac arrest) under a stereomicroscope (Leica Microsystems, S6E, GmbH, Wetzlar, Germany).

### 4.6. Statistical Analysis

Data were analyzed using Excel 2013 (Microsoft Corporation, Seattle, WA, USA) and GraphPad Prism 7 (GraphPad Software, Inc., La Jolla, CA, USA). Data are expressed as means ± standard error of mean (SEM), and groups were compared one-way ANOVA with Tukey’s post hoc test. *p*-values were considered significant at * *p* < 0.05, ** *p* < 0.01, and *** *p* < 0.001.

## 5. Conclusions

Our results suggest that neurochemical profiling-based approach could be used to better understand of anti-epileptic mechanism of GM-90432, potentially leading to new drug discovery and development of anti-seizure agents.

## Supplementary Materials

The following are available online at https://www.mdpi.com/1422-0067/22/3/1285/s1.
