# Supplementary material for: Neurochemical Effects of 4-(2Chloro-4-Fluorobenzyl)-3-(2-Thienyl)-1,2,4-Oxadiazol-5(4H)-One in the Pentylenetetrazole (PTZ)-Induced Epileptic Seizure Zebrafish Model
Source: Int J Mol Sci. 2021 Jan 28;22(3):1285. doi: 10.3390/ijms22031285 (PMC7865321; doi:10.3390/ijms22031285)

Supplementary Material

Neurochemical Effects of 4-(2Chloro-4-Fluorobenzyl)-3-(2-Thienyl)-1,2,4-Oxadiazol-5(4H)-One in the Pentylenetetrazole (PTZ)-Induced Epileptic Seizure Zebrafish Model

Seong Soon Kim [1,†], Hyemin Kan [1,†], Kyu-Seok Hwang [1], Jung Yoon Yang [1], Yuji Son [1], Dae-Seop Shin [1], Byung Hoi Lee [1], Se Hwan Ahn [2], Jin Hee Ahn [2], Sung-Hee Cho [3,*] and Myung Ae Bae [1,4,*]

[1] Bio & Drug Discovery Division, Korea Research Institute of Chemical Technology, Daejeon 34114, Korea; firstsay@krict.re.kr (S.S.K.); khm4029@krict.re.kr (H.K.); kshwang@krict.re.kr (K.-S.H.); yjy1608@krict.re.kr (J.Y.Y.); yjson@krict.re.kr (Y.S.); dsshin@krict.re.kr (D.-S.S.); bnhlee@krict.re.kr (B.H.L.)

[2] Department of Chemistry, Gwangju Institute of Science and Technology, Gwangju 61005, Korea; ansehwan@gist.ac.kr (S.H.A.); jhahn@gist.ac.kr (J.H.A.)

[3] Chemical Analysis Center, Korea Research Institute of Chemical Technology, Daejeon 34114, Korea

[4] Department of Medicinal Chemistry and Pharmacology, University of Science & Technology, Daejeon 34113, Korea

* Correspondence: shc0429@krict.re.kr (S.-H.C.); mbae@krict.re.kr (M.A.B.)

† These authors contributed equally on this work.

Table S1. Levels of neurochemicals in brain samples of zebrafish exposed to GM-90432, PTZ, or GM-90432 + PTZ including control group.

Neurochemicals		Concentration[a] (mean ± SD)			
		Controls (G1, n = 12)	GM-90432 (G2, n = 12)	PTZ (G3, n = 12)	GM-90432 + PTZ (G4, n = 12)
Amino acids	VAL	273 ± 44.7	344 ± 43.5	313 ± 39.8	358 ± 49.8
	PRO	611 ± 118	753 ± 247	714 ± 90.8	789 ± 102
	ALA	79751 ± 5396	86360 ± 5310	84935 ± 5345	84120 ± 8686
	ARG	2026 ± 219	2331 ± 387	2317 ± 337	2351 ± 299
	ASP	9159 ± 1419	6179 ± 881	4961 ± 1033	3434 ± 688
	HA	100 ± 8.16	101 ± 11.6	95.1 ± 10.8	97.4 ± 11.9
	HIS	27397 ± 3831	28082 ± 2399	28132 ± 3846	27150 ± 3309
	LYS	15571 ± 2391	21803 ± 1917	19180 ± 2739	22738 ± 3522
	LEU	528 ± 79.2	623 ± 60.0	595 ± 75.9	638 ± 104
	THRE	937 ± 185	1012 ± 304	1012 ± 181	972 ± 160
Cholinergic	ACHO	15.6 ± 1.65	14.4 ± 1.61	13.0 ± 0.78	13.2 ± 2.94
	CHO	5611 ± 597	5442 ± 602	7226 ± 972	6520 ± 744
	BET	5692 ± 1154	5516 ± 759	6068 ± 1319	5411 ± 680
	SE	2001 ± 350	2073 ± 412	2230 ± 321	2239 ± 359
Dopaminergic	PHE	751 ± 68.1	824 ± 72.6	888 ± 85.9	952 ± 82.7
	TYR	1152 ± 268	1185 ± 214	1378 ± 187	1369 ± 234
	DA	14.0 ± 1.31	17.0 ± 1.91	14.4 ± 1.55	16.3 ± 1.61
	NE	14.7 ± 2.13	15.3 ± 1.62	14.7 ± 2.24	14.7 ± 1.48
	NM	8.96 ± 1.62	7.71 ± 0.88	12.2 ± 2.06	9.91 ± 1.45
	3-MT	1152 ± 268	1185 ± 214	1378 ± 187	1369 ± 234
Serotonergic	TYRP	319 ± 30.8	367 ± 49.8	374 ± 51.7	397 ± 56.4
	5-HTP	3.43 ± 0.90	4.09 ± 1.54	2.85 ± 0.68	3.73 ± 0.99
	5-HT	10.3 ± 1.27	12.2 ± 2.00	8.51 ± 0.71	10.7 ± 1.40
	5-HIAA	45.0 ± 7.36	43.8 ± 6.01	51.8 ± 9.62	45.1 ± 7.08
	KYN	1.38 ± 0.90	1.02 ± 0.54	1.24 ± 1.22	1.16 ± 0.64
GABAergic	GABA	29418 ± 2235	26649 ± 2323	35507 ± 3976	31516 ± 3150
	GLU	38529 ± 2712	39816 ± 3252	38657 ± 3190	40388 ± 3359
	GLN	12898 ± 1929	18030 ± 1587	15954 ± 2201	18976 ± 2841

[a]Concentration was expressed as ng/g (mean ± SD); [b]NS, not significant

Table S2. Statistical analysis using ANOVA and Tukey test of neurotransmitters in brain samples of zebrafish exposed to GM-90432, PTZ, or GM-90432 + PTZ including control group.

Neurotransmitters		*P*-value using Tukey test						ANOVA
		G1 : G2	G1 : G3	G1 : G4	G2 : G3	G2 : G4	G3 : G4	
Amino acids	VAL	0.0018	0.1333	0.0002	0.3506	0.8658	0.0832	< 0.0001
	PRO	0.1221	0.3635	0.0327	0.9259	0.9371	0.6307	< 0.0398
	ALA	0.0661	0.2037	0.3435	0.9462	0.8234	0.9891	NS
	ARG	0.0998	0.1250	0.0706	0.9995	0.9986	0.9932	< 0.0480
	ASP	<0.0001	<0.0001	<0.0001	0.0311	<0.0001	0.0044	< 0.0001
	HA	0.9976	0.7061	0.9429	0.5903	0.8762	0.9553	NS
	HIS	0.9599	0.9512	0.9980	>0.9999	0.9070	0.8932	NS
	LYS	<0.0001	0.0110	<0.0001	0.0973	0.8318	0.0124	< 0.0001
	LEU	0.0306	0.1983	0.0095	0.8258	0.9703	0.5657	< 0.0093
	THRE	0.8244	0.8253	0.9768	>0.9999	0.9685	0.9688	NS
Cholinergic	ACHO	0.3844	0.0083	0.0167	0.3066	0.4453	0.9939	< 0.0054
	CHO	0.9441	<0.0001	0.0228	<0.0001	0.0050	0.1082	< 0.0001
	BET	0.9738	0.8002	0.9037	0.5466	0.9940	0.3948	NS
	SE	0.9620	0.4176	0.3835	0.7129	0.6762	>0.9999	NS
Dopaminergic	PHE	0.1114	0.0005	<0.0001	0.2058	0.0012	0.1897	< 0.0001
	TYR	0.9854	0.0867	0.1059	0.1759	0.2089	0.9997	< 0.0293
	DA	0.0002	0.9426	0.0079	0.0013	0.6333	0.0347	< 0.0001
	NE	0.8772	>0.9999	0.9997	0.8786	0.9125	0.9998	NS
	NM	0.2189	<0.0001	0.4504	<0.0001	0.0066	0.0038	< 0.0001
	3-MT	0.6605	<0.0001	<0.0001	<0.0001	<0.0001	0.9828	< 0.0293
Serotonergic	TYRP	0.0824	0.0360	0.0013	0.9843	0.4212	0.6381	< 0.0024
	5-HTP	0.4530	0.5557	0.9046	0.0359	0.8496	0.2042	< 0.0495
	5-HT	0.0103	0.0148	0.8933	<0.0001	0.0615	0.0020	< 0.0001
	5-HIAA	0.9787	0.1428	>0.9999	0.0613	0.9720	0.1545	NS
	KYN	0.7493	0.9825	0.9260	0.9213	0.9805	0.9946	NS
GABAergic	GABA	0.1242	<0.0001	0.3307	<0.0001	0.0015	0.0114	< 0.0001
	GLU	0.7475	0.9996	0.4753	0.8023	0.9700	0.5362	NS
	GLN	<0.0001	0.0071	<0.0001	0.1074	0.7154	0.0079	< 0.0001

Table S3. Levels of neurosteroids in brain samples of zebrafish exposed to GM-90432, PTZ, or GM-90432 + PTZ including control group.

**Steroids**	**Concentration[a] (mean ± SD)**			
	**Controls (G1, n = 6)**	**GM-90432 (G2, n = 6)**	**PTZ (G3, n = 6)**	**GM-90432 + PTZ (G4, n = 6)**
E2	0.26 ± 0.12	0.86 ± 0.14	0.11 ± 0.03	0.39 ± 0.13
T	0.014 ± 0.004	0.015 ± 0.007	0.015 ± 0.004	0.011 ± 0.004
DHT	0.007 ± 0.003	0.013 ± 0.005	0.004 ± 0.002	0.012 ± 0.003
C	0.02 ± 0.01	0.02 ± 0.01	0.20 ± 0.08	0.17 ± 0.11
Prog	5.45 ± 2.05	10.38 ± 3.02	2.53 ± 0.67	6.33 ± 1.30
5α-dihydroProg	5.13 ± 2.15	8.98 ± 3.37	2.93 ± 1.40	6.58 ± 1.62
Allo-P	4.38 ± 1.69	7.62 ± 1.60	2.40 ± 0.80	5.47 ± 2.54

[a]Concentration was expressed as ng/g (mean ± SD); [b]NS, not significant

Table S4. Statistical analysis using ANOVA and Tukey test of neurosteroids in brain samples of zebrafish exposed to GM-90432, PTZ, or GM-90432 + PTZ including control group.

Neurosteroids	*P*-value using Tukey test						ANOVA
	G1 : G2	G1 : G3	G1 : G4	G2 : G3	G2 : G4	G3 : G4	
E2	<0.0001	0.1260	0.1984	<0.0001	<0.0001	0.0015	< 0.0001
T	0.9685	0.9561	0.8313	>0.9999	0.5740	0.5401	NS
DHT	0.0339	0.4844	0.0575	0.0013	0.9939	0.0024	< 0.0005
C	>0.9999	0.0015	0.0095	0.0015	0.0097	0.8456	< 0.0002
Prog	0.0017	0.0794	0.8637	<0.0001	0.0096	0.0157	< 0.0001
5α-dihydroProg	0.0375	0.3600	0.6896	0.0009	0.2883	0.0513	< 0.002
Allo-P	0.0232	0.2433	0.7169	0.0003	0.1859	0.0329	< 0.0005

Figure S1. Protective effect of GM-90432 in survival test in zebrafish larvae. 5 dpf zebrafish larvae were treated GM-90432 in four concentrations with or without 5 mM PTZ (n=10). After 24 h, Survived larvae were counted and compared to the control group (non-treated larvae) at the percent level.

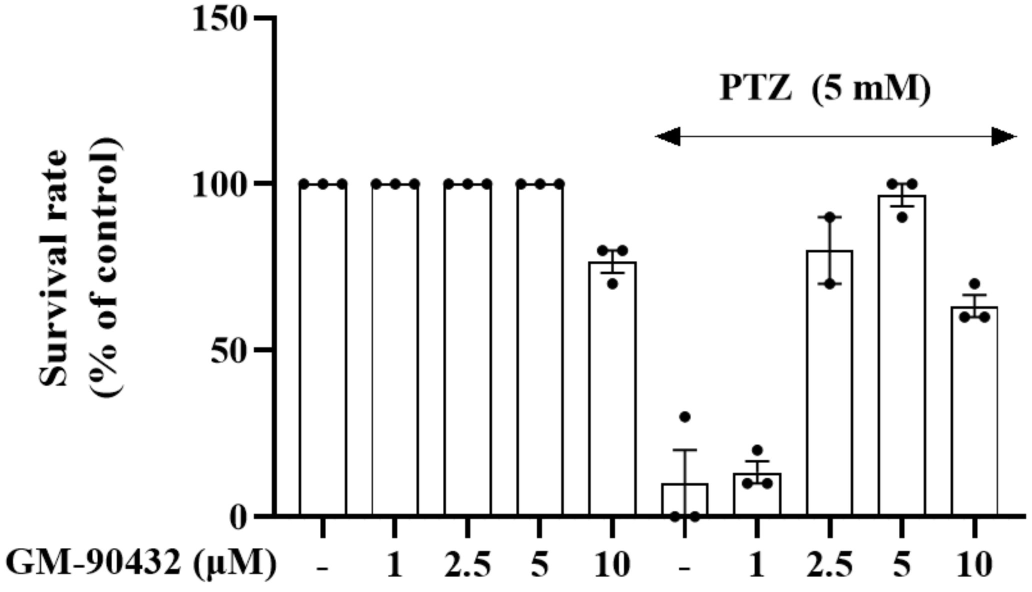

Supplement: Supplementary file 1 [file ijms-22-01285-s001.pdf]
